# Editorial: Investigations into the potential benefits of artificial intelligence and deep learning to surgical oncologists

**DOI:** 10.3389/fonc.2023.1243305

**Published:** 2023-07-11

**Authors:** Simon C. Williams, Hani J. Marcus

**Affiliations:** ^1^ Wellcome/EPSRC Centre for Interventional and Surgical Sciences, University College London, London, United Kingdom; ^2^ Department of Neurosurgery, St George’s University Hospital, London, United Kingdom; ^3^ Division of Neurosurgery, National Hospital for Neurology and Neurosurgery, London, United Kingdom

**Keywords:** artificial intelligence, machine learning, surgery, oncology, computer vision, deep learning

‘Any sufficiently advanced technology is indistinguishable from magic.”

So said science fiction writer Arthur C. Clarke, though few could have predicted the vast advances made in healthcare technology realised over the past decades. In the realm of surgical oncology, this sentiment holds true as we witness the rise of artificial intelligence (AI) and deep learning. These transformative technologies have the potential to revolutionise the field, enabling surgical oncologists to achieve remarkable advancements. This Research Topic aims to explore the potential benefits of AI and deep learning in assisting surgical oncologists and enhancing their decision-making capabilities. By harnessing the power of these technologies, we can aspire to achieve more accurate diagnoses, personalised treatment plans, optimised surgical interventions, and improved patient care (1).

This Research Topic presents nine publications spanning the surgical landscape, featuring research applied to Hepatobiliary Surgery (Jeong et al.; Li et al.; Huang et al.; Wang et al.), Orthopaedics (Yan et al.), Urology (Zhang et al.), Otolaryngology and Neurosurgery (Hill et al.; CRANIAL consortium). Each article is a demonstration of the unique opportunity AI poses to modern surgical oncologists. Here, we summarise the key insights presented in these publications, highlighting the advancements that AI presents in the field of surgical oncology.

First, the marriage of AI and deep learning with surgical oncology allows for intelligent data analysis on an unprecedented scale. These transformative technologies excel at swiftly and comprehensively processing vast amounts of complex data, as exemplified by the work of the CRANIAL Consortium et al. Their publication showcases machine learning-driven identification of predictors of cerebrospinal fluid rhinorrhoea following endonasal skull base surgery, utilising a vast corpus of surgical data. Yet AI driven data analysis is not confined to text data - within the domain of machine learning is computer vision, a branch of AI that gives computer platforms understanding of image and video data (2). Advances in this field, particularly in radiomics, have garnered global attention due to their remarkable progress; however, their application in the operating theatre has yet to be fully realised (3). Within this Research Topic, Wang et al. describe the construction of a survival prediction model that integrates multimodal imaging data with clinical data, showcasing how AI can lead to comprehensive insights and personalised treatment planning, optimising patient outcomes.

Second, the work published in this Research Topic presents how AI and deep learning techniques have the potential to enhance surgical guidance and assistance. Zhang et al. demonstrate the utility of computer vision in pre-operative planning and intraoperative decision making through their generation of a model able to predict renal perfusion regions based on automated segmentation of renovascular imaging. Such advancement stands to increase patient safety through ever-more precise surgical planning. The application of surgical AI is not confined to the pre- and post-operative stages; by analysing intraoperative data, such as live imaging, physiological signals, and surgical instrument tracking, AI algorithms can provide surgical oncologists with valuable guidance, assisting them in navigating critical structures, optimizing surgical margins, and ensuring precise tumour resection (4). Moreover, AI-powered systems can detect and predict potential complications, alerting surgeons in advance and allowing for timely interventions, ultimately leading to improved patient safety and outcomes (5).

Perhaps most valuable, however, is the promise of AI to offer predictive models that shape treatment decisions and outcomes, as demonstrated by numerous articles within this Research Topic. By leveraging large datasets, these models identify prognostic factors, predict treatment responses, and stratify patients into risk groups (6). Jeong et al. describe the creation survival prediction platform for patients with intrahepatic cholangiocarcinoma (ICC), enabling categorisation of patients into risk groups to guide clinical interventions. A similar prognostic approach is adopted by Yan et al., who describe the creation of a deep learning model to predict overall survival in chondrosarcoma patients. Notably, their DeepSurv model outperformed traditional models of survival prediction, highlighting the unique capability of machine learning methods to identify subtle relationships between variables in large, complex datasets. Applications such as this give an insight into how AI will individualise treatment decisions based on patient-specific data, replacing crude and generic risk prediction systems. Surgical oncologists can harness these insights to tailor treatment plans to individual patients, optimize the sequencing of therapies, and explore alternative strategies.

The integration of AI and deep learning transcends clinical practice and extends to surgical training and education. Virtual reality (VR) and augmented reality (AR) platforms, combined with AI algorithms, can simulate realistic surgical scenarios, providing surgical trainees with a safe and controlled environment for practicing complex procedures, such as tumour resections. By analysing trainee performance and offering real-time feedback, AI-powered systems may accelerate the learning curve, enhancing surgical skills acquisition (7). The scope of AI to benefit trainees does not stop there, however – AI algorithms may aid in curating educational resources, extracting key insights from scientific literature, and delivering personalised learning experiences to surgical oncologists. Hill et al. demonstrate the ability of AI to aid disease classification, through their use of AI to subclassify glioblastoma, a disease with a profoundly poor prognosis, enabling a clear taxonomy and better prediction of patient outcomes.

The articles presented within this Research Topic showcase the rich field of AI research in surgical oncology, yet whilst the potential benefits of AI and deep learning in surgical oncology are vast, ethical considerations and challenges must be navigated. Issues such as data privacy, algorithm bias, transparency, and accountability must be addressed to ensure patient safety, maintain trust in the healthcare system, and mitigate potential risks (2). Close collaboration between clinicians, researchers, policymakers, and regulatory bodies is crucial to establish guidelines and frameworks that uphold ethical standards and govern the integration of AI in surgical oncology (6).

We sincerely believe that the contents of this Research Topic will be of interest to surgeons, oncologists, and members of the wider healthcare team alike. AI and deep learning have the potential to revolutionise the field of surgical oncology, the true benefits of which are yet to be fully realised. By embracing the future, we embark on a journey to redefine healthcare.

## Author contributions

SW - writing - original draft. HM - writing - reviewing and editing; conceptualization. All authors contributed to the article and approved the submitted version.
